# Clinical validation of a machine-learned, point-of-care system to IDENTIFY pulmonary hypertension

**DOI:** 10.1183/23120541.01287-2024

**Published:** 2025-09-22

**Authors:** Dalton McLean, John Rommel, John A. Steuter, William S. Carroll, Mark Rabbat, Sudarshan Rajagopal, Venkatraman Srinivasan, Dean J. Kereiakes, Michael C. Roberts, Abhijit Raval, Navid Nemati, Farhad Fathieh, Timothy Burton, Horace R. Gillins, Ian Shadforth, Shyam Ramchandani, Charles R. Bridges, Vallerie V. McLaughlin

**Affiliations:** 1 LeBauer Cardiovascular Research Foundation, Greensboro, NC, USA; 2 Novant Health New Hanover Regional Medical Center, Wilmington, NC, USA; 3 Bryan Heart, Lincoln, NE, USA; 4 Cardiology Associates of North Mississippi, Tupelo, MS, USA; 5 Loyola University Medical Center, Maywood, IL, USA; 6 Duke University Medical Center, Durham, NC, USA; 7 Allegheny Health Network Research Institute, Pittsburgh, PA, USA; 8 The Lindner Research Center at The Christ Hospital, Cincinnati, OH, USA; 9 Lexington Medical Center Heart & Vascular, West Columbia, SC, USA; 10 AnMed Health, Anderson, SC, USA; 11 Analytics For Life, Inc, Toronto, ON, Canada; 12 CorVista Health, Inc., Bethesda, MD, USA; 13 University of Michigan Health, Ann Arbor, MI, USA

## Abstract

**Background:**

Pulmonary hypertension (PH) is a collection of diverse disorders, defined by mean pulmonary artery pressure (mPAP) ≥21 mmHg (most recent guidelines) or ≥25 mmHg (previous guidelines, that underpins the field's past work) measured by right heart catheterisation (RHC). Considering the difficulties in diagnosing PH and the subsequent treatment delays, there is a need for novel diagnostics to enable prompt detection.

**Methods:**

An algorithm to assess mPAP elevation was validated using subjects with elevated mPAP from RHC (positive cohort) and subjects with low probability of PH by stringent screening of transthoracic echocardiography (TTE) PH indicators (negative cohort). 25 mmHg and 21 mmHg were pre-specified as the co-primary and secondary sensitivity end-points, respectively, at 0.70. Specificity was the co-primary end-point at 0.60. The algorithm cut-point was pre-defined. The area under the receiver operator characteristic curve (ROC-AUC) was assessed at both mPAP thresholds.

**Findings:**

462 subjects were consecutively enrolled across 18 US clinical sites between August 2019 and September 2022. Sensitivity at 25 mmHg and 21 mmHg was 0.82 (95% CI 0.78–0.87) and 0.78 (95% CI 0.73–0.82), respectively, with specificity of 0.92 (95% CI 0.87–0.96), passing the study end-points. The ROC-AUC values at 25 mmHg and 21 mmHg were 0.95 (95% CI 0.93–0.96) and 0.93 (95% CI 0.91–0.95), respectively. Further, performance was similar across PH subgroups (pre-capillary, combined pre- and post-capillary, and isolated post-capillary), as well as between men and women.

**Interpretation:**

The algorithm's performance is comparable, or possibly superior to, TTE, given that the tricuspid regurgitant velocity is not measurable in up to 41% of TTE cases. The test is a stress-free, noninvasive front-line test, presenting advantages to patients, physicians and healthcare systems.

## Shareable abstract

**A machine-learned, point-of-care algorithm for pulmonary hypertension has been validated in a symptomatic population, yielding sensitivity of 82% and specificity of 92% – comparable to, or possibly superior to, transthoracic echocardiogram** https://bit.ly/3DbzKzt

## Introduction

Pulmonary hypertension (PH) encompasses a diverse group of disorders, defined by a mean pulmonary arterial pressure (mPAP) of ≥25 mmHg at rest, as confirmed by right heart catheterisation (RHC) [1]. Recent guidelines by the European Respiratory Society (ERS) and the European Society of Cardiology (ESC) in 2022 have lowered the definition of PH to an mPAP ≥21 mmHg [2]. The most commonly used clinical definition of PH, historically, as presented in the 2015 ESC/ERS guidelines, uses a disease threshold of mPAP≥25 mmHg [3]. The bulk of the scientific literature to date, including all the reports referenced in a 2019 meta-analysis of transthoracic echocardiography (TTE) performance, has focused on the threshold of ≥25 mmHg [4].

PH is most commonly found in individuals with left heart failure (systolic or diastolic), with prevalence estimates ranging from 25 to 83% [5, 6]. The prevalence of PH shows a strong and independent correlation with age [7]. PH is widespread, with an estimated prevalence of 1% in the global population and as much as 10% in individuals older than 65 [8]. Prognosis depends on access to appropriate medical care and on the World Symposium on Pulmonary Hypertension (WSPH) classification (groups 1–5). However, up to 39% of patients may fall into multiple groups [9–13], with overlap between groups 2 and 3 being the most common. PH resulting from left-sided heart disease (group 2) may have a 12-month mortality rate as high as 32% [9]. Early detection of PH allows for necessary interventions and therapies, offering the potential to alter the disease's progression, enhance survival and promote health equity.

The haemodynamic definitions of PH are shown in table 1. Pulmonary arterial hypertension (PAH) is WSPH group 1, an aggressive subtype that leads to right ventricular pressure and volume overload causing right ventricular failure and, in many cases, early death [10]. Unfortunately, PAH is often diagnosed at an advanced stage, when pathophysiologic changes have become irreversible, with 1-year mortality rates of 39% and 36% reported in US and French Registries, respectively [3, 11, 13]. Although there have been substantial advancements in understanding the cellular and genetic basis of PAH, the diagnostic delay has shown little improvement for about three decades [4, 12]. A multicentre retrospective study of PAH patients found that the mean±sd time from symptom onset to diagnosis was 47±34 months, requiring 5.3±3.8 primary care visits and evaluations by 3.0±2.1 subspecialists [14]. There is a similar average delay in the diagnosis of group 4 PH, namely chronic thromboembolic PH [15]. The impact of late diagnosis across all types of PH is that treatment options may become restricted due to comorbidities and organ damage, significantly compromising the patient's quality of life [16].

**TABLE 1:** Definition of pulmonary hypertension (PH) and subgroups

	2015 ESC/ERS guidelines [3]	2022 ESC/ERS guidelines [2]
**PH**	↑ mPAP ≥25 mmHg	↑ mPAP ≥21 mmHg
**Pre-capillary PH**	↑ mPAP ≥25 mmHg ↓ PCWP ≤15 mmHg ↑ PVR >3 WU	↑ mPAP ≥21 mmHg ↓ PCWP ≤15 mmHg ↑ PVR >2 WU
**Post-capillary PH**	↑ mPAP ≥25 mmHg ↑ PCWP >15 mmHg ↓ PVR ≤3 WU	↑ mPAP ≥21 mmHg ↑ PCWP >15 mmHg ↓ PVR ≤2 WU
**Combined pre- and post-capillary PH**	↑ mPAP ≥25 mmHg ↑ PCWP >15 mmHg ↑ PVR >3 WU	↑ mPAP ≥21 mmHg ↑ PCWP >15 mmHg ↑ PVR >2 WU

ERS: European Respiratory Society; ESC: European Society of Cardiology; PCWP: pulmonary capillary wedge pressure; PVR: pulmonary vascular resistance; WU: Wood units.

The present standard of care requires the physician to have a suspicion of PH and that TTE is readily available. TTE tricuspid regurgitant velocity (TRV) measurement is required to achieve high-probability classification for PH [2]; however, unfortunately, TRV may be unmeasurable in a significant portion of patients. Establishing a precise rate of TRV immeasurability is difficult and beyond the scope of the present work. It depends on factors such as year of data collection, cohort composition and measurement setting (academic *versus* typical clinical environment). For instance, TRV immeasurability is disproportionately impacted in specific subgroups, such as those with COPD [17]. However, TRV immeasurability has been reported to be as high as 41% [18], with values of 16% [19], 29% [20] and 32% [21] also described. Note that in the absence of TRV, other echocardiographic indicators may enable a classification of at most intermediate probability of PH. However, even under ideal conditions (with TRV available), TTE still has a low positive predictive value (PPV). Specifically, an extensive meta-analysis of 29 studies with patients who underwent both TTE and RHC reported a TTE sensitivity of 83% and specificity of 72% [18]. Given a 6% PH prevalence [22], the corresponding PPV is 15.9%, with a strong negative predictive value (NPV) of 98.5%.

Artificial intelligence may play a valuable role in identifying patients with PH. For instance, DuBrock *et al*. [22] recently described an ECG-based neural network to detect PH; while the algorithm had a robust sensitivity in the overall group with dyspnoea (92% in the primary clinical site and 89% in the secondary test site), sensitivity was significantly reduced in critical subgroups. Specifically, pre-capillary PH sensitivity was 70% and 69%, and isolated post-capillary PH was 73% and 72%, in the primary and secondary sites, respectively. We believe it is possible to develop a methodology that maintains robust disease detection in these important subgroups.

Herein we describe the performance validation of a novel point-of-care diagnostic, the CorVista System, with a PH add-on. The primary assessment used a threshold of mPAP ≥25 mmHg, given the popularity of this definition, as discussed above. However, given the recent changes in the definition of PH, our secondary end-point utilised the definition of PH as an mPAP ≥21 mmHg. Reporting adhered to the STARD (Standards for Reporting of Diagnostic Accuracy Studies) guidelines [23] (supplementary appendix section 1).

## Methods

The IDENTIFY (NCT03864081) and IDENTIFY-PH (NCT04031989) trials, approved by the Western Institutional Review Board, are ongoing, multicenter, prospective, nonrandomised and selectively blinded studies. These repository studies are designed to gather physiological signals and associated subject metadata from individuals presenting with cardiovascular symptoms. Both studies are available on ClinicalTrials.gov (https://clinicaltrials.gov/study/NCT03864081 and https://clinicaltrials.gov/study/NCT04031989). This data was gathered for the development, refinement, testing and validation of machine-learned algorithms. All subjects were required to provide informed consent. Race and ethnicity were self-reported. Supplementary appendix section 2 contains the inclusion and exclusion criteria. The validation population comprised subjects who were consecutively enrolled from August 2019 to September 2022 (n=462) across 18 clinical sites (supplementary appendix section 3). No subjects included in the validation population were accessible to algorithm developers, nor were they involved in the algorithm development process.

### Signal acquisition device

**FIGURE 1:**
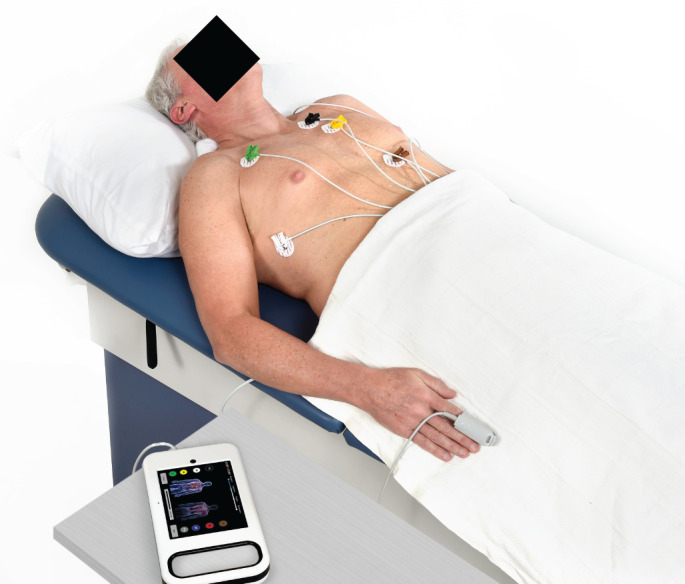
Demonstration of the signal acquisition, comprising seven electrodes (two not pictured, on the back and left ribs), as well as finger probe for photoplethysmogram.

A proprietary signal acquisition device recorded the orthogonal voltage gradient (OVG) using electrodes positioned on the thorax, generating three bipolar channels, namely left–right, top–bottom and front–rear. At the same time, the device collected photoplethysmogram (PPG) data *via* a finger probe. The patient remained in a supine position and at rest during the recording. The acquisition set-up is illustrated in figure 1. Signals were captured at a sampling rate of 8 kHz for 210 s and packaged with a patient identifier (study-specific), along with patient weight, height, date of birth and birth gender.

### Machine-learned algorithm

The development of the machine-learned algorithm was described previously [24]. In brief, the machine-learned algorithm for detecting PH was constructed using 217 features extracted from the OVG and PPG signals. Random Forest and Elastic Net models were trained to associate feature values with PH status, with outputs averaged in an ensemble. Three families of features accounted for 90% of the feature importance. The OVG conduction family contributed most significantly, capturing characteristics of the myocardial conduction pathway, including its variability. Repolarisation was the next family by significance, assessing myocardial recovery on the OVG through power distribution, morphology, variation and timing. The third family, respiration, assessed using both OVG and PPG, estimates the respiration waveform and characterises that estimation. The final algorithm was integrated into a high-throughput processing system, incorporating the cut-point that differentiates test-negativity from test-positivity, and was subsequently used to process the validation dataset.

### Population groups for validation

The co-primary end-points for algorithm validation were based on two distinct test groups, as follows: Population A, which was used to assess sensitivity, and Population B, which was used to assess specificity. Together, these groups formed a composite population consisting of subjects with elevated mPAP measurements from RHC (the gold standard to confirm mPAP elevation) and subjects with very low risk of having PH, as determined by stringent screening for indicators of PH using TTE. All patients were new to the care pathway and presented with symptoms of cardiovascular disease including shortness of breath, chest pain and exercise intolerance.

#### Population A: sensitivity test group

Population A comprised IDENTIFY-PH subjects who were referred for RHC, including the following:

1) Population A1: subjects with known elevated mPAP (≥25 mmHg) at RHC (primary end-point)
2) Population A2: subjects with known elevated mPAP (≥21 mmHg) at RHC (secondary end-point)

#### Population B: specificity test group

Population B consisted of IDENTIFY group 4 subjects, who had undergone coronary computed tomography angiography (CCTA) and were assessed by an independent core laboratory (Global Institute for Research, Midlothian, VA) to be negative for coronary artery disease. Specifically, these subjects had a CAD-RADS (Coronary Artery Disease–Reporting and Data System) score between 0 and 2, with no recommendation for additional testing nor follow-up. Subjects had also undergone TTE within 90 days of signal acquisition. They were required to have a low probability of PH, as determined by the ESC/ERS guidelines [2], for echocardiographic interpretation, where the initial criterion mandates a TRV of <2.8 m·s^−1^ or for the TRV to be unmeasurable. In addition, the TTE was negative for diastolic dysfunction based on the American Society of Echocardiography recommendations [25].

### Null hypotheses for validation

A clinically useful test should detect a large percentage of subjects that have the condition. The current front-line test (TTE) has a lower confidence bound (LCB) of 0.73 in the subjects in which it can be used (at minimum 59% of the incoming population) [18]. A new test with a similar LCB that can be used across the entire population would therefore improve on the current clinical workflow. Consequently, the LCB for sensitivity in detecting elevated mPAP among subjects in Populations A1 and A2 was set at 0.70, with an estimated point performance of 0.80. The use of subjects determined to have low probability of elevated mPAP by TTE is very close to the intended use population. The lower confidence bound for specificity of the TTE test is 0.53 when compared to RHC [18]. Setting the LCB for the new test at 0.60 and an estimated point performance of 0.75 is therefore reasonable. Given that the validation assesses two independent end-points, namely sensitivity and specificity, achieving at least 80% overall power requires that each end-point be individually powered at 90%. Assuming a sensitivity of 0.80, with a one-sided α value of 0.025 and 90% power, a minimum of 196 PH-positive subjects is necessary. Similarly, assuming a specificity of 0.75 under the same conditions, at least 102 PH-negative subjects are required. The hypotheses are as follows.

### Sensitivity

To evaluate sensitivity in confirming presence of PH within a symptomatic population (validation Population A), the hypothesis framework was defined as follows: H0 (null hypothesis) is sensitivity ≤0.70, while H1 (alternative hypothesis) is sensitivity >0.70. Validation Populations A1 and A2 were composed exclusively of PH-positive subjects confirmed *via* RHC after initial symptomatic presentation, making them directly applicable for sensitivity assessment. A normal approximation test was employed, utilising both the sensitivity estimate and its variance estimate, to assess the null hypothesis (that the true sensitivity is at or below the performance goal) against the alternative hypothesis that it exceeds this threshold. This analysis is conducted with a one-sided α=0.025, which is statistically equivalent to demonstrating that the lower bound of the two-sided 95% lower confidence interval surpasses the performance goal. Let n denote the sample size of PH-positive subjects and Sn denote the estimated sensitivity, then:

Estimateofvarianceofestimateofsensitivity=(Sn)(1−Sn)/n

### Specificity

To assess specificity in confirming the absence of significant PH in a symptomatic population (validation Population B), the hypothesis framework is defined as follows: H0 (null hypothesis) is specificity ≤0.60 and H1 (alternative hypothesis) is specificity >0.60. The calculation of specificity requires only those subjects who are definitively classified as PH-negative. A normal approximation test was utilised to evaluate the null hypothesis that the true specificity is at or below the performance goal against the alternative hypothesis that it is greater than the performance goal, conducted using a one-sided α=0.025. Let n represent the sample size for PH-negative subjects and Sp denote the estimated specificity, then:

Estimateofvarianceofestimateofspecificity=Sp(1−Sp)/n

### Further statistics and methodology

Signal acquisition took place before RHC (Population A) and before CCTA (Population B), although the TTE was prior to signal acquisition. To qualify for inclusion, subjects were required to fulfil all inclusion criteria, meet none of the exclusion criteria, undergo their initial reference test (CCTA or RHC) within 7 days of signal acquisition, not have any major protocol deviations and produce a signal that successfully passes quality checks. Failure to satisfy any of these conditions resulted in subject exclusion. Neither RHC nor TTE results were accessible to those responsible for invoking the score generation, nor was the score available to clinical investigators. The pairing of the algorithm scores and RHC/TTE results was conducted only by an independent third-party statistician (Technomics).

Beyond the co-primary end-points of sensitivity and specificity described, additional analyses included the assessment of the receiver operator characteristic curve and the area under the curve (ROC-AUC) and likelihood ratios (LRs). Subgroups evaluation, based on age, sex, PH subgroups and other typical criteria, was performed using sensitivity, specificity and the ROC-AUC. An analysis of the continuum of the score was performed by segmenting into tertiles.

### Role of the funding source

The study sponsor designed the study and provided the raw data to the independent statistician for analysis. All authors, including those employed by the sponsor, decided to submit the manuscript for publication.

## Results

There were 681 patients in Population A (supplementary appendix section 4A). Within this cohort, 3.7% (n=25) exhibited a major protocol deviation, which included cases where catheterisation occurred more than 7 days after signal acquisition. Additionally, 1% (n=7) did not have mPAP available from the RHC. Among the cohort, 350 subjects (53.9%) had an mPAP <25 mmHg, while 14 subjects (2.2%) did not have a recorded signal. The absence of a signal was attributed to insufficient time for signal acquisition prior to RHC, connectivity failures or improper device storage, such as failure to maintain an adequate charge or ensure an active internet connection. The remaining 285 subjects who were enrolled in Population A1 successfully met all inclusion criteria and no exclusion criteria without major protocol deviations. However, among these, 16.1% (n=46) did not achieve an acceptable signal quality. The remaining subjects (Population A1, sensitivity) were used for PH validation at the ≥25 mmHg end-point, n=239, table 2. As documented in supplementary appendix section 4B, there were 315 patients in Population A2 used for PH validation at the ≥21 mmHg end-point. The distribution of mPAP is shown in supplementary appendix section 5.

**TABLE 2:** Demographics and cardiac history

	Population A1	Population A2	Population B	Total
**Description**	mPAP ≥25 mmHg	mPAP ≥21 mmHg	TTE negatives	
**Number of subjects (N)**	239	315	147	462
**Age (years)**				
Mean±sd	66.7±11.6	67.5±11.7	55.8±11.6	63.8±12.9
<65	40.2 (96/239)	37.5 (118/315)	71.4 (105/147)	48.3 (223/462)
≥65	59.8 (143/239)	62.5 (197/315)	28.6 (42/147)	51.7 (239/462)
**Sex**				
Female	47.3 (113/239)	47.0 (148/315)	70.7 (104/147)	54.5 (252/462)
Male	52.7 (126/239)	53.0 (167/315)	29.3 (43/147)	45.5 (210/462)
**Ethnicity**				
Not Hispanic or Latino	97.9 (234/239)	98.1 (309/315)	99.3 (146/147)	98.5 (455/462)
Hispanic or Latino	1.3 (3/239)	1.3 (4/315)	0.7 (1/147)	1.1 (5/462)
Unknown	0.8 (2/239)	0.6 (2/315)	0.0 (0/147)	0.4 (2/462)
**Race**				
American Indian or Alaska Native	0.0 (0/239)	0.0 (0/315)	0.0 (0/147)	0.0 (0/462)
Asian	0.0 (0/239)	0.3 (1/315)	2.0 (3/147)	0.9 (4/462)
Black or African American	17.2 (41/239)	15.9 (50/315)	20.4 (30/147)	17.3 (80/462)
Native Hawaiian or other Pacific Islander	0.0 (0/239)	0.0 (0/315)	0.0% (0/147)	0.0 (0/462)
White/Caucasian	80.8 (193/239)	81.6 (257/315)	75.5 (111/147)	79.7 (368/462)
Other	1.3 (3/239)	1.6 (5/315)	1.4 (2/147)	1.5 (7/462)
Prefer not to answer	0.8 (2/239)	0.6 (2/315)	0.7 (1/147)	0.6 (3/462)
**BMI (kg·m^−2^)**				
Mean±sd	33.3±9.0	32.7±8.5	31.7±7.3	32.3±8.2
<30	44.4 (106/239)	44.8 (141/315)	49.7 (73/147)	46.3 (214/462)
≥30	55.6 (133/239)	55.2 (174/315)	50.3 (74/147)	53.7 (248/462)
<35	65.7 (157/239)	67.0 (211/315)	69.4 (102/147)	67.7 (313/462)
≥35	34.3 (82/239)	33.0 (104/315)	30.6 (45/147)	32.3 (149/462)
**Tobacco use**				
Never	40.2 (96/239)	41.6 (131/315)	58.5 (86/147)	47.0 (217/462)
Past or present	59.8 (143/239)	58.4 (184/315)	41.5 (61/147)	53.0 (245/462)
**Family history of heart attack**	26.8 (64/239)	27.0 (85/315)	41.5 (61/147)	31.6 (146/462)
** **Unknown	12.6 (30/239)	12.4 (39/315)	0.7 (1/147)	8.7 (40/462)
**Hypertension^#^**	82.8 (198/239)	82.2 (259/315)	56.5 (83/147)	74.0 (342/462)
**Diabetes^#^**	39.7 (95/239)	36.8 (116/315)	15.0 (22/147)	29.9 (138/462)
**Hypercholesterolemia/hyperlipidaemia^#^**	72.0 (172/239)	73.3 (231/315)	53.1 (78/147)	66.9 (309/462)
**COPD**	22.2 (53/239)	20.0 (63/315)	2.0 (3/147)	14.3 (66/462)

Data are presented as % (n/N), unless stated otherwise. BMI: body mass index; TTE: transthoracic echocardiogram. ^#^: One subject in population B did not have known hypertension, diabetes nor hypercholesterolemia/hyperlipidaemia status.

There were 170 patients in Population B (supplementary appendix section 6). In this group, 10.6% (n=18) had a major protocol deviation. Further, two (1.2%) did not have signal data (due to the same reasons as in Population A). The final 150 subjects in Population B successfully satisfied all inclusion and exclusion criteria without any major protocol deviations. Among these, 2% (n=3) failed to achieve acceptable signal quality. The remaining subjects (Population B, specificity) were used for PH validation, n=147 (table 2).

No adverse events were reported in Populations A or B.

**FIGURE 2:**
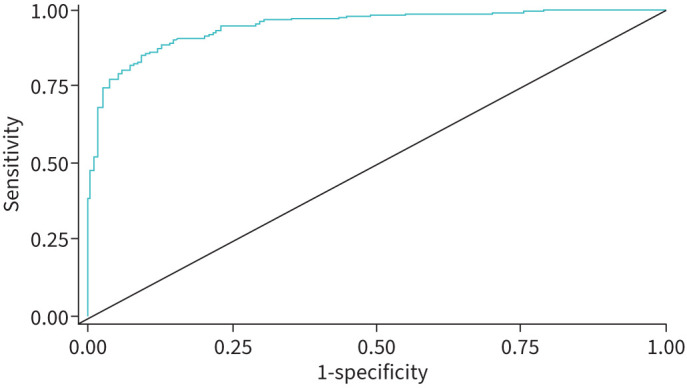
Receiver operating characteristic curve for the algorithm on the positive cohort (pulmonary hypertension (PH) defined by 2015 guidelines [3], with a mean pulmonary arterial pressure ≥25 mmHg) and negative cohort (symptomatic subjects negative for PH and diastolic dysfunction on transthoracic echocardiogram), yielding an area under the curve of 0.946 (95% CI 0.929–0.963).

As illustrated in table 3, the null hypothesis is rejected for both sensitivity and specificity. Consequently, the algorithm meets the predetermined primary and secondary end-points at the 95% confidence level. At the 25 mmHg end-point, the sensitivity was 0.82 (95% CI 0.78–0.87) and at the 21 mmHg end-point, the sensitivity was 0.78 (95% CI 0.73–0.82). Specificity was 0.92 (95% CI 0.87–0.96) for both end-points. The 2×2 tables can be found in supplementary appendix section 7. With a disease prevalence of 6%, as was found across multiple large institutional datasets of dyspnoeic patients [22], the PPV is 40% and the NPV is 99%. The algorithm ROC-AUC on the validation data for the primary end-point was 0.95 (95% CI 0.93–0.96), with the ROC curve presented as figure 2. Table 4 demonstrates strong performance for both men and women, and across the PH subgroups isolated post-capillary, pre-capillary and combined post- and pre-capillary PH, at both the end-points at 21 mmHg and 25 mmHg. The subgroup performances for the primary end-point can be found in supplementary appendix section 8, encompassing sex, body mass index (at both 30 and 35), age, diabetes, hypertension, hyperlipidaemia, tobacco use, COPD and ethnicity, which showed significant differences (p<0.01) in only the diabetes subgroups (both sensitivity and specificity). However, the overall performance between the diabetes subgroups is similar, with the diabetic subgroup exhibiting elevated sensitivity at lower specificity and the nondiabetic subgroup exhibiting elevated specificity and at lower sensitivity.

**TABLE 3:** Algorithm performance

Statistic	Pre-specified target	Value	p-value (greater than target)
**Sensitivity (25 mmHg)**	0.70	0.824 (95% CI 0.776–0.873)	<0.0001
**Sensitivity (21 mmHg)**	0.70	0.775 (95% CI 0.728–0.821)	0.0008
**Specificity**	0.60	0.918 (95% CI 0.874–0.963)	<0.0001
**ROC-AUC (25 mmHg)**	NA	0.946 (95% CI 0.929–0.963)	NA
**ROC-AUC (21 mmHg)**	NA	0.927 (95% CI 0.907–0.947)	NA

AUC: area under the curve; NA: not applicable; ROC: receiver operating characteristic.

**TABLE 4:** Pulmonary hypertension (PH) subgroup performance

PH defined by 2015 ESC/ERS guidelines [3] (mPAP ≥25 mmHg)				
	All subjects with PH	Pre-capillary	Combined pre- and post-capillary	Isolated post-capillary
**Both genders**				
Subgroup, n	239	54	29	42
ROC-AUC	0.946	0.963	0.991	0.961
Sensitivity	0.824	0.889	1.000	0.833
Specificity	0.918 (n=147)			
**Male**				
n	113	28	14	27
ROC-AUC	0.988	0.990	1.000	0.991
Sensitivity	0.833	0.821	1.000	0.889
Specificity	1.000 (n=43)			
**Female**				
n	126	26	15	15
ROC-AUC	0.923	0.973	0.985	0.935
Sensitivity	0.814	0.962	1.000	0.733
Specificity	0.885 (n=104)			

PH defined by 2015 ESC/ERS guidelines [3] (mPAP ≥25 mmHg)				
	All subjects with PH	Pre-capillary	Combined pre- and post-capillary	Isolated post-capillary
**Both genders**				
** **Subgroup, n	315	91	45	32
** **ROC-AUC	0.927	0.947	0.981	0.947
** **Sensitivity	0.775	0.846	0.933	0.813
** **Specificity	0.918 (n=147)			
**Male**				
** **n	167	44	23	21
** **ROC-AUC	0.977	0.985	1.000	0.981
** **Sensitivity	0.790	0.818	0.957	0.810
** **Specificity	1.000 (n=43)			
**Female**				
** **n	148	47	22	11
** **ROC-AUC	0.902	0.937	0.966	0.956
** **Sensitivity	0.757	0.872	0.909	0.818
** **Specificity	0.885 (n=104)			

AUC: area under the curve; ERS: European Respiratory Society; ESC: European Society of Cardiology; mPAP: mean pulmonary artery pressure; NA: not applicable; ROC: receiver operating characteristic.

The study was multicentre, but since enrolment gating was used across sites with ranges of volumes, subject contribution by site varied. Thus, it is reasonable to perform a sensitivity analysis of the highest enrolling site as compared to the others. The highest enrolling site had sensitivity and specificity of 86% and 93%, as compared to 81% and 89% at the other sites (p=0.43 and 0.34, respectively). Therefore, no significant difference in performance was observed from the highest enrolling site as compared to the other sites.

Segmenting the algorithm's test-negative scores into tertiles revealed a pattern in which lower negative LRs occurred in score ranges further away from zero as compared to closer to zero (supplementary appendix section 9). Inversely, segmenting the algorithm's test-positive scores segmentation exhibited higher positive LRs further away from zero as compared to closer to zero (supplementary appendix section 9).

Information on test repeatability and reproducibility is available in supplementary appendix section 10.

## Discussion

The results were derived from a large, blinded cohort consisting of 315 subjects that underwent RHC and 147 subjects that underwent TTE. For comparison, the PROMISE study was based on 10 003 patients presenting with cardiovascular symptoms and only 10% (1015) underwent left heart catheterisation (LHC) [26] and significantly fewer underwent RHC. The sensitivity calculation requires only patients that truly have PH; by definition, the RHC cohort encompasses all patients who can be identified as such when adhering to the standard of care.

The specificity calculation requires only patients that truly do not have PH. We estimate that ∼99% of patients presenting with cardiovascular symptoms who are negative for PH never undergo RHC, given that RHC is performed far less frequently than LHC [27, 28]. Accordingly, the specificity cohort, derived from TTE, represents the expected intended use population, specifically, patients exhibiting symptoms of cardiovascular disease but without prior evidence suggestive of significant PH. TTE is an excellent test to *rule out* PH when the ERS/ESC guidelines are applied, limiting the population to patients who are “low probability” for PH, with an estimated 99% negative predictive value for PH. The predefined end-points were designed to establish performance that is on par with or exceeding that of TTE as a PH diagnostic. For the PH definition of mPAP ≥25 mmHg, with point performances for sensitivity and specificity of 0.82 and 0.92, respectively, and lower confidence bounds that passed the primary end-point, this validation has confirmed that the algorithm is suitable for the intended use population, achieving performance comparable to or exceeding that of TTE. In particular, at the same disease prevalence of 6%, the algorithm has a PPV of 40%, as compared to TTE at 16%, while having similar NPVs of approximately 99%.

Historically, cardiology has seen an under-representation of women and minority groups [29]. The presented findings indicate comparable overall performance between males and females, and Black or African American subjects *versus* others. The findings further indicate comparable overall performance between diabetic and nondiabetic individuals, although diabetic subjects exhibited statistically significantly higher sensitivity and significantly lower specificity compared to nondiabetic subjects. Notably, the algorithm performance remains consistent across age groups, a critical characteristic given that certain PH subtypes exhibit distinct age and sex biases. For instance, PAH is more prevalent among younger females, whereas other types, such as isolated post capillary PH, are more commonly observed in individuals over the age 65. Further, no difference in sensitivity was found between subjects with and without COPD, which is complementary to TTE, in that TTE suffers from a reduced sensitivity to PH in COPD patients given higher rates of TRV immeasurability. Thus, the algorithm may provide additive diagnostic value alongside TTE in cases such as this where TTE may be inconclusive.

When evaluating the change in probability of disease in using this algorithm, the LRs are LR+=10.05 and LR−=0.19 at 25 mmHg and LR+=9.45 and LR−=0.25 at 21 mmHg. Additionally, the algorithm's continuous output allows for a more nuanced interpretation of test results beyond the binary output. As noted, the negative LR decreases in more strongly negative score ranges, while the positive LR increases in higher positive score ranges. Consequently, post-test disease probability assessment can be refined based on a more granular spectrum of scores rather than a simple dichotomous outcome.

Evaluating the performance of machine-learned algorithms requires careful consideration of both intrinsic and extrinsic biases, which may inadvertently become ingrained within the algorithm [29]. Frequent challenges include variability in reference standard methodology between institutions and regional differences in disease prevalence. The validation population includes regions with higher prevalence of PH (Colorado, Virginia, Illinois, North Carolina, Ohio), moderate (Florida, South Carolina) and lower prevalence comparatively (Texas, Nebraska, Louisiana) [30]. Thus, the validation population is expected to be representative of the intended use population, with data collected from 18 distinct sites to further mitigate bias.

Another common challenge in machine learning is overfitting [29]. The results reported here originate from a blinded validation set, enrolled consecutively after training was completed. Differences in site locations and personnel between the training and validation datasets further reinforce confidence that the findings are generalisable to the intended use population.

We were delighted to discover that the algorithm's AUC, sensitivity and specificity remained consistently comparable across all PH subtypes, namely pre-capillary, combined pre- and post-capillary, and isolated post capillary PH. This suggests that our test effectively addressed the limitation of the existing methodology [22], pending direct comparison within the same dataset. This finding is extremely important as treatments have now been approved for PAH (group 1, pre-capillary PH) and PH secondary to pulmonary disease (group 3, pre-capillary PH). The drug sotatercept demonstrated exceptional efficacy as a treatment for PAH in its pivotal phase 3 trial and has received approval from the US Food and Drug Administration [31]. Moreover, sotatercept is in an ongoing trial (CADENCE, NCT04945460, https://clinicaltrials.gov/study/NCT04945460) for the treatment of a much larger group of patients with combined pre-and post-capillary PH. In addition, SGLT2 (sodium–glucose cotransporter 2) inhibitors received a new class 2a recommendation for the treatment of heart failure with preserved ejection fraction (HFpEF) in the 2022 American Heart Association/American College of Cardiology/Heart Failure Society of America heart failure guidelines [32]. HFpEF is the most common cause of group 2 PH. The introduction of these highly effective new treatments for PH substantially enhances the public health advantages of early detection across all PH subtypes.

The inability to provide a test result in 16% of patients is a limitation of the device, though this rate is comparable to that of magnetic resonance imaging, which has been shown to exhibit significant motion artifacts in 7.5% of outpatients [33]. However, we are working to decrease this rate through training and minor device enhancements. A limitation of our study is the underrepresentation of Hispanic patients; although this group makes up 16% of the US population [34], they comprised only 1.1% of our study cohort. However, this is in alignment with a National Institutes for Health report that found that Hispanic patients comprise only about 1% of clinical trial participants in the US, perhaps due to language barriers and socioeconomic factors such as reduced access to healthcare [34].

### Conclusions

In conclusion, the algorithm performance presented is arguably comparable or superior to TTE for the identification of patients with PH. Unlike TTE, the current standard of care noninvasive test to assess the presence or absence of PH, the results are available prior to the patient leaving the office, minimising the fraction lost to follow-up. In rural areas, access to TTE is hindered by travel constraints, scheduling challenges and apprehension. As a result, many rural patients never undergo TTE (Tracy Neal, Cullman Regional Medical Group, Cullman, AL, USA; personal communication). Even when performed, up to 41% of cases fail to complete the PH assessment due to the inability to measure the TRV [18], which may partly explain the lower life expectancy observed among residents of rural regions in the US. In this study, the algorithm was validated in a cohort representative of the intended use population, specifically, symptomatic patients with no previous diagnosis of PH. Notably, overall performance is consistent between female and male subjects and does not vary across different age groups. This system fulfils the need for a nonstress, noninvasive, front-line test for PH, offering substantial benefits to the patient, physician and the broader healthcare system.

## Data Availability

Relevant de-identified subsets of the dataset may be shared with academic investigators on a case-by-case basis.

## Supplementary material

**Please note:** supplementary material is not edited by the Editorial Office, and is uploaded as it has been supplied by the author. Supplementary material 01287-2024.SUPPLEMENT

## References

[1] Dunlap B, Weyer G. Pulmonary hypertension: diagnosis and treatment. Am Fam Physician 2016; 94: 463–469. 27637122

[2] Humbert M, Kovacs G, Hoeper MM, et al. 2022 ESC/ERS guidelines for the diagnosis and treatment of pulmonary hypertension: developed by the task force for the diagnosis and treatment of pulmonary hypertension of the European Society of Cardiology (ESC) and the European Respiratory Society (ERS). Eur Heart J 2022; 43: 3618–3731. doi:10.1093/eurheartj/ehac237 36017548

[3] Galiè N, Humbert M, Vachiery JL, et al. 2015 ESC/ERS guidelines for the diagnosis and treatment of pulmonary hypertension: the joint task force for the diagnosis and treatment of pulmonary hypertension of the European Society of Cardiology (ESC) and the European Respiratory Society (ERS): endorsed by: Association for European Paediatric and Congenital Cardiology (AEPC), International Society for Heart and Lung Transplantation (ISHLT). Eur Heart J 2016; 37: 67–119. doi:10.1093/eurheartj/ehv317 26320113

[4] Ni JR, Yan PJ, Liu SD, et al. Diagnostic accuracy of transthoracic echocardiography for pulmonary hypertension: a systematic review and meta-analysis. BMJ Open 2019; 9: e033084. doi:10.1136/bmjopen-2019-033084 PMC6937087 31871259

[5] Guazzi M, Borlaug BA. Pulmonary hypertension due to left heart disease. Circulation 2012; 126: 975–990. doi:10.1161/CIRCULATIONAHA.111.085761 22908015

[6] Vachiéry JL, Adir Y, Barberà JA, et al. Pulmonary hypertension due to left heart diseases. J Am Coll Cardiol 2013; 62: D100–D108. doi:10.1016/j.jacc.2013.10.033 24355634

[7] Lam CS, Borlaug BA, Kane GC, et al. Abstract 6206: age-associated increases in pulmonary artery systolic pressure in the general population. Circulation 2008; 118: Suppl. 18, S_1157–S_1158. doi:10.1161/circ.118.suppl_18.S_1157-d PMC2753443 19433755

[8] Hoeper MM, Humbert M, Souza R, et al. A global view of pulmonary hypertension. Lancet Respir Med 2016; 4: 306–322. doi:10.1016/S2213-2600(15)00543-3 26975810

[9] Farber HW, Gibbs S. Under pressure: pulmonary hypertension associated with left heart disease. Eur Respir Rev 2015; 24: 665–673. doi:10.1183/16000617.0059-2015 26621980 PMC9487627

[10] Voelkel NF, Quaife RA, Leinwand LA, et al. Right ventricular function and failure: report of a National Heart, Lung, and Blood Institute working group on cellular and molecular mechanisms of right heart failure. Circulation 2006; 114: 1883–1891. doi:10.1161/CIRCULATIONAHA.106.632208 17060398

[11] Humbert M, Sitbon O, Chaouat A, et al. Pulmonary arterial hypertension in France: results from a national registry. Am J Respir Crit Care Med 2006; 173: 1023–1030. doi:10.1164/rccm.200510-1668OC 16456139

[12] Hassoun PM. Pulmonary arterial hypertension. N Engl J Med 2021; 385: 2361–2376. doi:10.1056/NEJMra2000348 34910865

[13] Brown LM, Chen H, Halpern S, et al. Delay in recognition of pulmonary arterial hypertension: factors identified from the REVEAL registry. Chest 2011; 140: 19–26. doi:10.1378/chest.10-1166 21393391 PMC3198486

[14] Strange G, Gabbay E, Kermeen F, et al. Time from symptoms to definitive diagnosis of idiopathic pulmonary arterial hypertension: the delay study. Pulm Circ 2013; 3: 89–94. doi:10.4103/2045-8932.109919 23662179 PMC3641745

[15] Gall H, Hoeper MM, Richter MJ, et al. An epidemiological analysis of the burden of chronic thromboembolic pulmonary hypertension in the USA, Europe and Japan. Eur Respir Rev 2017; 26: 160121. doi:10.1183/16000617.0121-2016 28356407 PMC9488926

[16] Mathai SC, Ghofrani HA, Mayer E, et al. Quality of life in patients with chronic thromboembolic pulmonary hypertension. Eur Respir J 2016; 48: 526–537. doi:10.1183/13993003.01626-2015 27076580 PMC4967564

[17] Celeski M, Segreti A, Polito D, et al. Traditional and advanced echocardiographic evaluation in chronic obstructive pulmonary disease: the forgotten relationship. Am J Cardiol 2024; 217: 102–118. doi:10.1016/j.amjcard.2024.02.022 38412881

[18] Janda S, Shahidi N, Gin K, et al. Diagnostic accuracy of echocardiography for pulmonary hypertension: a systematic review and meta-analysis. Heart 2011; 97: 612–622. doi:10.1136/hrt.2010.212084 21357375

[19] Gall H, Yogeswaran A, Fuge J, et al. Validity of echocardiographic tricuspid regurgitation gradient to screen for new definition of pulmonary hypertension. EClinicalMedicine 2021; 34: 100822. doi:10.1016/j.eclinm.2021.100822 33997731 PMC8102717

[20] O'Leary JM, Assad TR, Xu M, et al. Lack of a tricuspid regurgitation Doppler signal and pulmonary hypertension by invasive measurement. J Am Heart Assoc 2018; 7: e009362. doi:10.1161/JAHA.118.009362 29960993 PMC6064901

[21] Garcia-Ribas C, Ble M, Gómez M, et al. Importance of tricuspid regurgitation velocity threshold in risk assessment of pulmonary hypertension-long-term outcome of patients submitted to aortic valve replacement. Front Cardiovasc Med 2021; 8: 720643. doi:10.3389/fcvm.2021.720643 34859063 PMC8631497

[22] DuBrock HM, Wagner TE, Carlson K, et al. An electrocardiogram-based AI algorithm for early detection of pulmonary hypertension. Eur Respir J 2024; 64: 2400192. doi:10.1183/13993003.00192-2024 38936966 PMC11269769

[23] Bossuyt PM, Reitsma JB, Bruns DE, et al. STARD 2015: an updated list of essential items for reporting diagnostic accuracy studies. Clin Chem 2015; 61: 1446–1452. doi:10.1373/clinchem.2015.246280 26510957

[24] Nemati N, Burton T, Fathieh F, et al. Pulmonary hypertension detection non-invasively at point-of-care using a machine-learned algorithm. Diagnostics (Basel) 2024; 14: 897. doi:10.3390/diagnostics14090897 38732312 PMC11083349

[25] Nagueh SF, Smiseth OA, Appleton CP, et al. Recommendations for the evaluation of left ventricular diastolic function by echocardiography: an update from the American Society of Echocardiography and the European Association of Cardiovascular Imaging. Eur J Echocardiogr 2016; 17: 1321–1360. doi:10.1016/j.echo.2016.01.011 27422899

[26] Douglas PS, Hoffmann U, Patel MR, et al. Outcomes of anatomical *versus* functional testing for coronary artery disease. N Engl J Med 2015; 372: 1291–1300. doi:10.1056/NEJMoa1415516 25773919 PMC4473773

[27] Grymuza M, Małaczyńska-Rajpold K, Jankiewicz S, et al. Right heart catheterization procedures in patients with suspicion of pulmonary hypertension–experiences of a tertiary center. Postepy Kardiol Interwencyjnej 2017; 13: 295–301. doi:10.5114/aic.2017.71610 29362571 PMC5770859

[28] Yeo KK, Maddox TM, Carey E, et al. Right-and left-sided heart catheterization as a quality marker for catheterization laboratories (from the National Veterans Affairs clinical assessment reporting and tracking program). Am J Cardiol 2014; 114: 1758–1762. doi:10.1016/j.amjcard.2014.08.047 25316348

[29] Tat E, Bhatt DL, Rabbat MG. Addressing bias: artificial intelligence in cardiovascular medicine. Lancet Digit Health 2020; 2: e635–e636. doi:10.1016/S2589-7500(20)30249-1 33328028

[30] Hyduk A, Croft JB, Ayala C, et al. Pulmonary hypertension surveillance – United States, 1980–2002. Surveillance Summaries 2004; 54: 1–28. 16280974

[31] Hoeper MM, Badesch DB, Ghofrani HA, et al. Phase 3 trial of sotatercept for treatment of pulmonary arterial hypertension. N Engl J Med 2023; 388: 1478–1490. doi:10.1056/NEJMoa2213558 36877098

[32] Heidenreich PA, Bozkurt B, Aguilar D, et al. 2022 AHA/ACC/HFSA guideline for the management of heart failure: executive summary: a report of the American College of Cardiology/American Heart Association Joint Committee on Clinical Practice Guidelines. J Am Coll Cardiol 2022; 79: 1757–1780. doi:10.1016/j.jacc.2021.12.011 35379504

[33] Andre JB, Bresnahan BW, Mossa-Basha M, et al. Toward quantifying the prevalence, severity, and cost associated with patient motion during clinical MR examinations. J Am Coll Radiol 2015; 12: 689–695. doi:10.1016/j.jacr.2015.03.007 25963225

[34] National Institutes for Health. The precision medicine initiative cohort program – building a research foundation for 21st century medicine. Bethesda, National Institutes for Health, 2015.

