# Supplementary material for: Clinical validation of a machine-learned, point-of-care system to IDENTIFY pulmonary hypertension
Source: ERJ Open Res. 2025 Sep 22;11(5):01287-2024. doi: 10.1183/23120541.01287-2024 (PMC12451580; doi:10.1183/23120541.01287-2024)
Supplement: Supplementary file 1 [file 01287-2024.SUPPLEMENT.pdf]

Supplementary Appendix Section 1 – STARD checklist

Section & Topic	No	Item	Reported on page #
**TITLE OR ABSTRACT**			
	**1**	Identification as a study of diagnostic accuracy using at least one measure of accuracy (such as sensitivity, specificity, predictive values, or AUC)	1
**ABSTRACT**			
	**2**	Structured summary of study design, methods, results, and conclusions (for specific guidance, see STARD for Abstracts)	2
**INTRODUCTION**			
	**3**	Scientific and clinical background, including the intended use and clinical role of the index test	4-5
	**4**	Study objectives and hypotheses	5
**METHODS**			
Study design	**5**	Whether data collection was planned before the index test and reference standard were performed (prospective study) or after (retrospective study)	5
Participants	**6**	Eligibility criteria	Supplement Section 2
	**7**	On what basis potentially eligible participants were identified (such as symptoms, results from previous tests, inclusion in registry)	6-7
	**8**	Where and when potentially eligible participants were identified (setting, location and dates)	5, Supplement Section 3
	**9**	Whether participants formed a consecutive, random or convenience series	5
Test methods	**10a**	Index test, in sufficient detail to allow replication	5-6
	**10b**	Reference standard, in sufficient detail to allow replication	6-7
	**11**	Rationale for choosing the reference standard (if alternatives exist)	6
	**12a**	Definition of and rationale for test positivity cut-offs or result categories of the index test, distinguishing pre-specified from exploratory	6
	**12b**	Definition of and rationale for test positivity cut-offs or result categories of the reference standard, distinguishing pre-specified from exploratory	4-6
	**13a**	Whether clinical information and reference standard results were available to the performers/readers of the index test	8
	**13b**	Whether clinical information and index test results were available to the assessors of the reference standard	8

Analysis	**14**	Methods for estimating or comparing measures of diagnostic accuracy	7-8
	**15**	How indeterminate index test or reference standard results were handled	8
	**16**	How missing data on the index test and reference standard were handled	8
	**17**	Any analyses of variability in diagnostic accuracy, distinguishing pre-specified from exploratory	7-8
	**18**	Intended sample size and how it was determined	7
**RESULTS**			
Participants	**19**	Flow of participants, using a diagram	Supplement Sections 4 & 6
	**20**	Baseline demographic and clinical characteristics of participants	Table 2
	**21a**	Distribution of severity of disease in those with the target condition	Supplement Section 5
	**21b**	Distribution of alternative diagnoses in those without the target condition	Table 2
	**22**	Time interval and any clinical interventions between index test and reference standard	9
Test results	**23**	Cross tabulation of the index test results (or their distribution) by the results of the reference standard	Supplement Section 7
	**24**	Estimates of diagnostic accuracy and their precision (such as 95% confidence intervals)	Table 3
	**25**	Any adverse events from performing the index test or the reference standard	9
**DISCUSSION**			
	**26**	Study limitations, including sources of potential bias, statistical uncertainty, and generalisability	11
	**27**	Implications for practice, including the intended use and clinical role of the index test	11-12
**OTHER INFORMATION**			
	**28**	Registration number and name of registry	5
	**29**	Where the full study protocol can be accessed	5
	**30**	Sources of funding and other support; role of funders	9

Supplementary Appendix Section 2 – Inclusion/Exclusion Criteria

<table>
<tr><th>Inclusion Criteria</th><th>Exclusion Criteria</th></tr>
<tr><td>1. Patients ≥ 18 years old
2. Cardiovascular symptoms
3. Scheduled to undergo either right heart catheterization (IDENTIFY-PH), or computed tomographic angiography (IDENTIFY Group 4)
4. Ability to understand the requirements of the study and to provide written informed consent
5. Normal Sinus Rhythm (NSR) at time of phase signal collection (study procedure)</td><td rowspan="6">1. Prior heart valve replacement or repair
2. Infiltrative myocardial disease (amyloid, sarcoid, right ventricular dysplasia)
3. Presence of cardiac implantable electronic device (CIED), including implantable cardioverter defibrillator (ICD), pacemaker (PM), implantable loop recorders and other monitors
4. Implantable Neuro-stimulators
5. Congenital Heart Disease
6. Pregnant or breast feeding
7. Currently taking any Type IA, IC or III antiarrhythmics
8. Any history of amiodarone use
9. Clinically significant chest deformity (e.g., pectus excavatum or pectus carinatum)
10. Breast implants
11. Neuromuscular disease if the condition results in tremor or muscle fasciculations</td></tr>
<tr><th>Exclusion Criteria – IDENTIFY-PH Only</th></tr>
<tr><td>Prior lung or heart transplant</td></tr>
<tr><th>Exclusion Criteria – IDENTIFY Group 4 Only</th></tr>
<tr><td>1. Prior documented history of myocardial infarction (MI)
2. Suspected acute myocardial infarction (AMI) at current presentation
3. Prior coronary artery bypass grafting (CAGB)
4. Previous sustained or paroxysmal atrial or ventricular arrythmia</td></tr>
</table>

Supplementary Appendix Section 3 – Clinical Sites

Clinical Site	Location	N
**LeBauer Cardiovascular Research Foundation (The Moses H. Cone Memorial Hospital d/b/a Cone Health)**	Greensboro, NC	178
**New Hanover Regional Medical Center**	Wilmington, NC	72
**Bryan Heart**	Lincoln, NE	35
**Austin Heart**	Austin, TX	17
**Sentara Hospital and Medical Group**	Norfolk, VA	11
**Lexington Center Medical Heart and Vascular**	West Columbia, SC	5
**Jackson Heart Clinic**	Jackson, MS	5
**Cardiology Associates Research, LLC**	Tupelo, MS	29
**Duke University**	Durham, NC	16
**AnMed Health**	Anderson, SC	17
**Louisiana State University Health Sciences Center – New Orleans**	New Orleans, LA	1
**Allegheny Health Network Research Institute**	Pittsburgh, PA	15
**The Lindner Research Center at The Christ Hospital**	Cincinnati, OH	8
**UC-MHS (UCHealth Memorial)**	Colorado Springs, CO	11
**Loyola University Medical Center**	Chicago, IL	24
**Clearwater Cardiovascular Consultants – Clearwater**	Clearwater, FL	14
**Clearwater Cardiovascular Consultants – Morton Plant Hospital**	Clearwater, FL	3
**Clearwater Cardiovascular Consultants – Mease Countryside**	Safety Harbor, FL	1
		**462**

Supplementary Appendix Section 4A – CONSORT Flow for Population A1 (25mmHg)

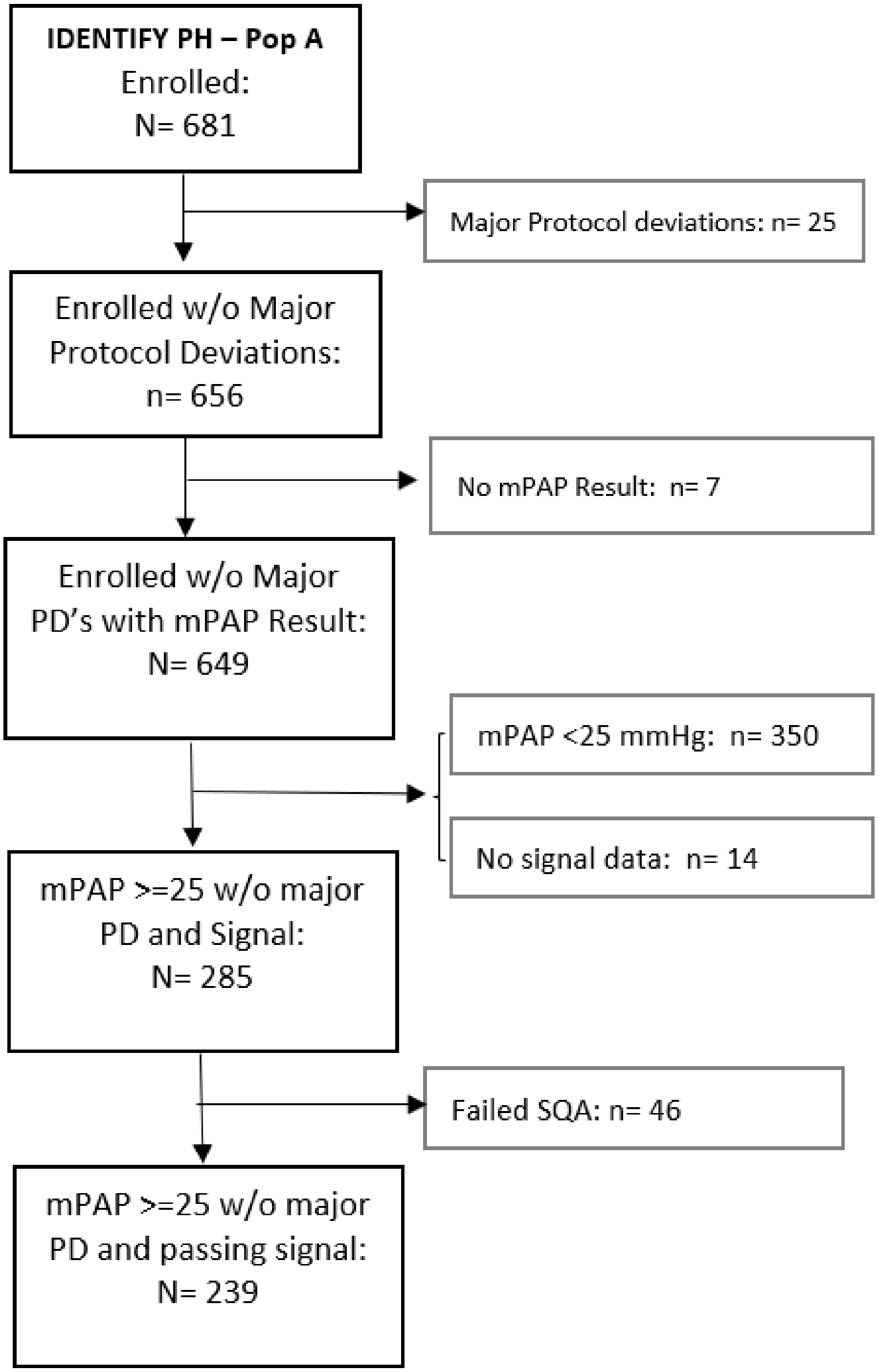

Supplementary Appendix Section 4B – CONSORT Flow for Population A2 (21mmHg)

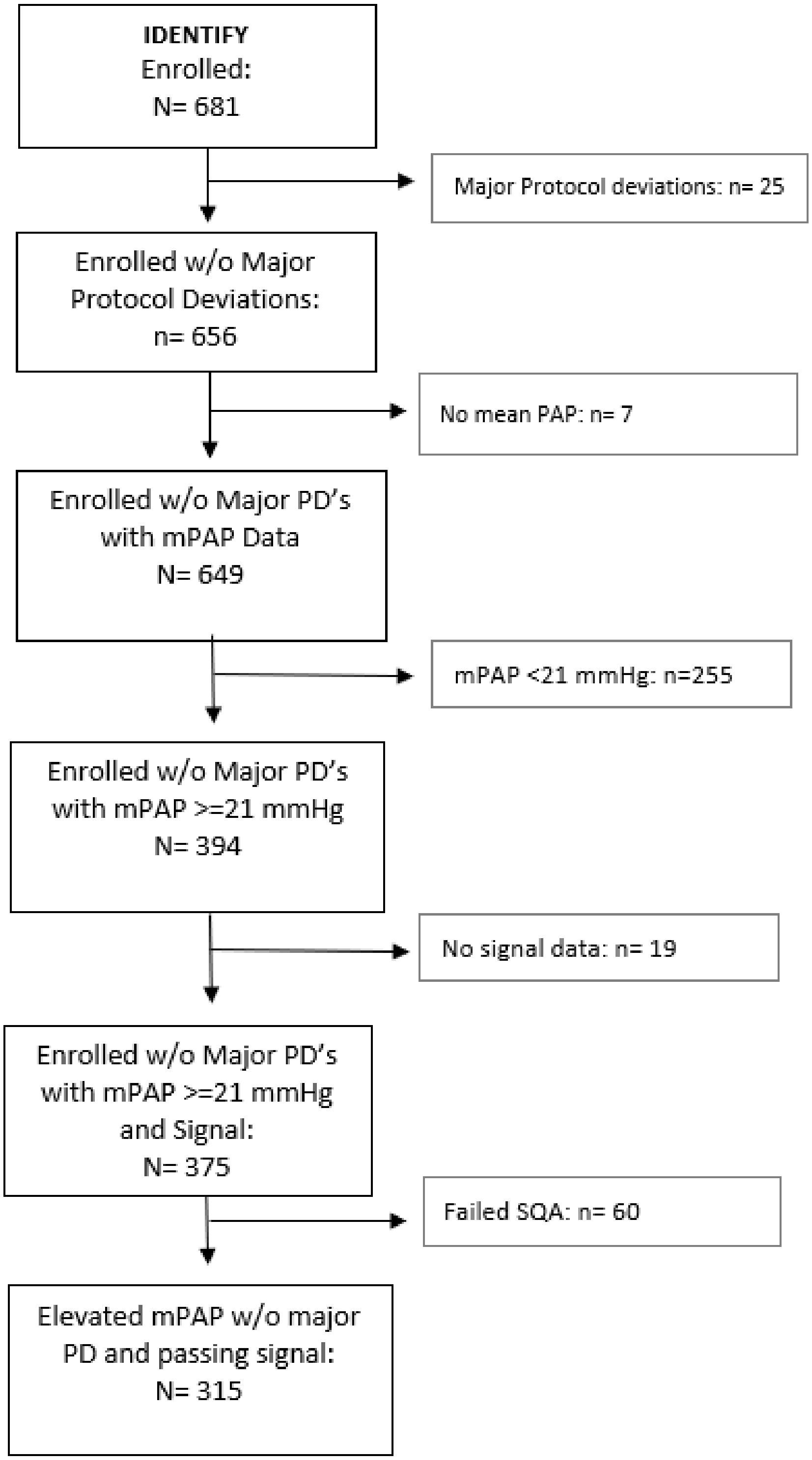

Supplementary Appendix Section 5 – Distribution of mPAP

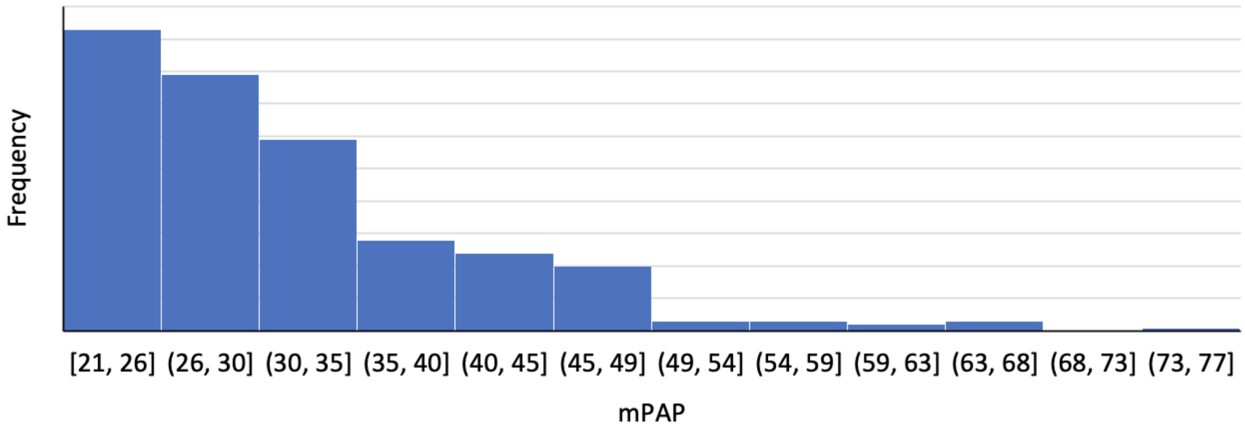

Supplementary Appendix Section 6 – CONSORT Flow for Population B

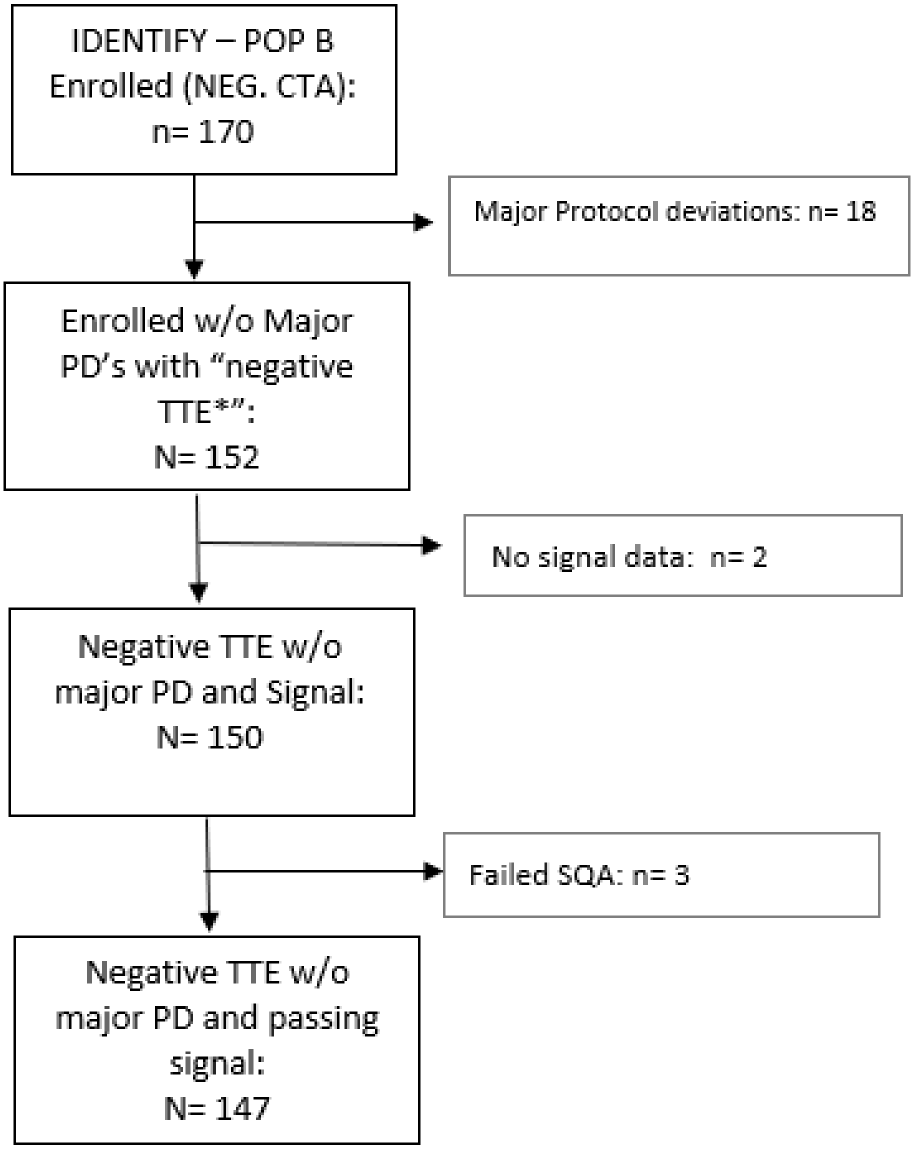

Supplementary Appendix Section 7 – 2x2 Tables

	Predicted PH+	Predicted PH-	Performance
True PH+ (25mmHg)	197	42	Sensitivity: 82.4%
True PH+ (21mmHg)	244	71	Sensitivity: 77.5%
True PH-	12	135	Specificity: 91.8%

Supplementary Appendix Section 8 – Subgroup Performance

Subgroup	Sensitivity (n/N)	95% CI	p-value	Specificity (n/N)	95% CI	p-value
Female	0.814 (92/113)	(0.742, 0.886)	0.7003	0.885 (92/104)	(0.823, 0.946)	0.0194
Male	0.833 (105/126)	(0.768, 0.898)		1.000 (43/43)	(1.000, 1.000)	
BMI ≥ 30	0.812 (108/133)	(0.746, 0.878)	0.5723	0.905 (67/74)	(0.839, 0.972)	0.5638
BMI < 30	0.840 (89/106)	(0.770, 0.909)		0.932 (68/73)	(0.874, 0.989)	
BMI ≥ 35	0.793 (65/82)	(0.705, 0.880)	0.3538	0.844 (38/45)	(0.739, 0.950)	0.0210
BMI < 35	0.841 (132/157)	(0.784, 0.898)		0.951 (97/102)	(0.909, 0.993)	
Age ≥ 65	0.804 (115/143)	(0.739, 0.869)	0.3195	0.905 (38/42)	(0.816, 0.994)	0.7035
Age < 65	0.854 (82/96)	(0.784, 0.925)		0.924 (97/105)	(0.873, 0.975)	
Diabetic	0.905 (86/95)	(0.846, 0.964)	0.0074	0.773 (17/22)	(0.598, 0.948)	0.0060
Non-Diabetic	0.771 (111/144)	(0.702, 0.839)		0.944 (118/125)	(0.904, 0.984)	
Hypertensive	0.823 (163/198)	(0.770, 0.876)	0.9269	0.892 (74/83)	(0.825, 0.958)	0.1761
Non-Hypertensive	0.829 (34/41)	(0.714, 0.944)		0.953 (61/64)	(0.901, 1.005)	
Hyperlipidemic	0.814 (140/172)	(0.756, 0.872)	0.5000	0.897 (70/78)	(0.830, 0.965)	0.3245
Non-Hyperlipidemic	0.851 (57/67)	(0.765, 0.936)		0.942 (65/69)	(0.887, 0.997)	
COPD	0.849 (45/53)	(0.753, 0.945)	0.5909	n/a*		
No COPD	0.817 (152/186)	(0.762, 0.873)				
Tobacco User**	0.825 (118/143)	(0.763, 0.887)	0.9683	0.902 (55/61)	(0.827, 0.976)	0.5331
Non-Tobacco User**	0.823 (79/96)	(0.747, 0.899)		0.930 (80/86)	(0.876, 0.984)	
White/Caucasian	0.808 (156/193)	(0.753, 0.864)	0.1503	0.937 (104/111)	(0.892, 0.982)	0.0695
Black or African American	0.902 (37/41)	(0.812, 0.993)		0.833 (25/30)	(0.700, 0.967)	

* Specificity cannot be assessed in COPD subgroups due to the low prevalence in this population (2%)

** Tobacco use is past or present, vs. never

Supplementary Appendix Section 9 – Likelihood Ratio by Score Range

PH Defined with mPAP≥25mmHg				
PH Test Result	Tertile	Score Range	Tertile Likelihood Ratio	Overall Likelihood Ratio
Negative	Q1	(-0.489, -0.325)	0.05	0.19
	Q2	(-0.324, -0.175)	0.13	
	Q3	(-0.169, -0.012)	0.36	
Positive	Q1	(0.004, 0.175)	4.88	10.05
	Q2	(0.178, 0.331)	13.20	
	Q3	(0.333, 0.460)	not calculable*	
PH Defined with mPAP≥21mmHg				
PH Test Result	Tertile	Score Range	Tertile Likelihood Ratio	Overall Likelihood Ratio
Negative	Q1	(-0.489, -0.316)	0.08	0.25
	Q2	(-0.315, -0.160)	0.21	
	Q3	(-0.156, -0.007)	0.41	
Positive	Q1	(0.004, 0.170)	4.60	9.45
	Q2	(0.171, 0.311)	12.35	
	Q3	(0.313, 0.460)	not calculable*	

*No false positives in this tertile from which to calculate the likelihood ratio, therefore it technically equates to infinity. Practically, it is high, but the magnitude of the value is unknown.

Supplementary Appendix Section 10 – Test Repeatability and Reproducibility

8 study subjects underwent 5 serial tests with the same operator to assess device repeatability, and ANOVA testing showed an insignificant p-value for differences between the resultant test scores.

Similarly, 19 study subjects underwent 3 serial tests with different operators to assess device reproducibility, and ANOVA testing showed an insignificant p-value for differences between the resultant test scores. Further, the intraclass correlation coefficient (ICC, Type 1) was 0.87 (95% CI: 0.74-0.94); the value is high, indicating good to excellent reliability between the repeated measurements[*].

[*] Koo, Terry K., and Mae Y. Li. "A guideline of selecting and reporting intraclass correlation coefficients for reliability research." Journal of chiropractic medicine 15.2 (2016): 155-163
